# Position sensitive measurement of trace lithium in the brain with NIK (neutron-induced coincidence method) in suicide

**DOI:** 10.1038/s41598-021-86377-x

**Published:** 2021-03-25

**Authors:** J. Schoepfer, R. Gernhäuser, S. Lichtinger, A. Stöver, M. Bendel, C. Delbridge, T. Widmann, S. Winkler, M. Graw

**Affiliations:** 1 Institute of Legal Medicine, Ludwig-Maximilian University, Munich, Germany; 2 Department of Physics, Technical University of Munich, Munich, Germany; 3 Institute of General and Surgical Pathology of the Technical University of Munich, Munich, Germany

**Keywords:** Biophysics, Neuroscience, Medical research

## Abstract

Mood disorder is the leading intrinsic risk factor for suicidal ideation. Questioning any potency of mood-stabilizers, the monovalent cation lithium still holds the throne in medical psychiatric treatment. Furthermore, lithium`s anti-aggressive and suicide-preventive capacity in clinical practice is well established. But little is still known about trace lithium distribution and any associated metabolic effects in the human body. We applied a new technique (neutron-induced coincidence method “NIK”) utilizing the ^6^Li(n,α)^3^H reaction for the position sensitive, 3D spatially resolved detection of lithium traces in post-mortem human brain tissue in suicide versus control. NIK allowed, for the first time in lithium research, to collect a three dimensional high resolution map of the regional *trace* lithium content in the non lithium-medicated human brain. The results show an anisotropic distribution of lithium, thus indicating a homeostatic regulation under physiological conditions as a remarkable link to essentiality. In contrast to suicide we could empirically prove significantly higher endogenous lithium concentrations in white compared to gray matter as a general trend in non-suicidal individuals and lower lithium concentrations in emotion-modulating regions in suicide.

## Introduction

Every 20–40 s a person dies due to suicide, proving a stable phenomenon with a worldwide average of nearly 1 Million suicides per year. The rate of attempted suicides and—thus possibly severe consequences—may be up to 20 times higher, which means one attempt every 1–2 s in average worldwide^1,2^ and is expected to increase in the current SARS-CoV-2-pandemic. As suicide is indubitable a complex multifactorial event and based on interactions in between genetic, environmental and psychosocial factors^3^, one of the leading risk factors for suicidal ideation is psychiatric disease^4^. CAVANAGH et al. showed in a systematic review of psychological autopsy studies, that 9 of 10 suicide victims were prior diagnosed with a mental disorder, mostly depression^5^. Mood disorders in general come with up to 10 times higher lifetime suicide risk compared to the non-psychiatric population and the risk in bipolar disorder is up to 30 times higher^6^.

Since numerous decades now, lithium salts represent not only the gold standard in acute and long-term maintenance management of mania and depressive disorders^7–9^. Lithium`s anti-aggressive and suicide-preventive capacity in clinical practice is well established^10–12^. Numerous ecological studies indicate similar effects of nutritional lithium^13,14^, with ranges up to several mg Li^+^/d^15^.

SHEARD et al. reported for the first time in the beginning of the 1970s, that lithium in pharmacological doses is accompanied by a significant reduction in aggressive behavior^16–18^. Furthermore lithium`s antisuicidal potency seems undeniable. A potential link between lithium-medication and antisuicidal effects was first questioned in 1972^19^ and statistically proven in numerous studies in the following decades^20–22^. Lately there has been increasing evidence for the hypothesis, that naturally occurring lithium may have a modulating protective effect on suicide rates too. As early as 1970 conducted epidemiological studies already questioned a potential impact of naturally occurring lithium e.g. in tap water on mental health^23,24^. Two decades later SCHRAUZER proved a significant correlation between tap water lithium content and the prevalence of suicides in the catchment area^25^. In the last decade there has been a broader international approach reevaluating the results by conducting similar studies^26–33^, further confirming the postulate of trace lithium’s antisuicidal capacity.

Despite the largely banal fact, that lithium is ingested daily by food and acts as a permanent resident in the human body in analytically relevant concentrations, little is known about possible beneficial effects on human health or any physiological function. While blood serum concentrations close to toxic limits in psychiatric pharmacotherapy show lithium`s well proven remarkable efficiency in equilibration of mood cycles, although based on only sparse amounts of experimental data in literature^34^, any impact of subclinical or trace serum concentrations is still in doubt. Tap or mineral water, grains and vegetables are main sources for daily lithium intake. Tap water lithium concentrations can range in between few µg/l and several mg/l worldwide^15^. Considering usual therapeutic medical doses in bipolar disorder treatment, the lithium intake via oral administration of lithium salts in psychiatric practice like lithium carbonate or lithium orotate ranges within approx. 80 mg Li^+^/oral unit and 250 mg Li^+^/d. This dose is associated with well proven therapeutic efficacy, but contrasted by a narrow therapeutic range, a potential risk of intoxication and the necessity of close drug monitoring during lithium treatment. On the other hand, the daily dietary Li^+^-intake can be estimated to be in the order of up to several mg Li^+^-ions. This means, that depending on the environment, the trace lithium uptake ranges a factor 100 lower or even less compared to daily pharmacological doses. Therefore, a total trace lithium human body content of several mg would be in the same range as essential trace elements like cobalt or selenium. However, in spite of lithium’s comparable much lower atomic weight, under physiological conditions the number of reactive Li^+^-ions in the human body can thus be estimated up to 20 times higher than cobalt, up to 4 times higher than selenium and a factor 10^3^ lower than the alkaline earth metal magnesium (Mg^++^), a gap which in turn is fully compensated in pharmacotherapy.

Being marked as the most ignoble of precious metals, with only 1 valence electron the Li^+^-cation is highly reactive. Lithium´s charge density is similar to calcium (Ca^++^) and in spite of it`s much lower weight lithium`s ionic radius is almost identical to Mg^++^^35^. It is well known that the ions of sodium, potassium, calcium and magnesium dominate neurotransmission and signal transduction processes. Lithium is the only monovalent alkali cation with the ability to replace sodium in stabilizing the nerve’s resting membrane potential. It also influences potassium regulated enzymatic processes^36^. Bringing into discussion a more fundamental sight of the microscopic neurotransmission, which goes much beyond the Hodgkin–Huxley-Model^37^, it sounds therefore not surprising, that lithium with its remarkable electrochemical properties might influence enzymatic processes linked to neuropsychiatric disorders as a sufficient competitor^38–40^. But despite being one of the most researched elements with proven essentiality in mammals^15^, it is still not fully clear, how lithium may change metabolic systems in favour of protective emotional stabilisation. Being the smallest and lightest solid element, its ability to carry out processes in biological organisms may only be explained at molecular levels and thus make any research into the biological profile of the “magic ion” comparably difficult^35^.

There is still a lack in studies on any physiological relevance of trace lithium levels in human brain. Though currently there is still no consistent detailed picture how lithium acts inside the human body. In this context of particular interest is, how endogenous lithium is distributed in the brain, a question to which, in lack of a suitable method, an answer has hitherto been reserved.

Measurement of the lithium concentration with established methods like atomic absorption spectroscopy (AAS) or inductively coupled plasma mass spectrometry (ICP-MS) need macroscopic samples disintegrated in advance and do not permit detailed anatomical information. In vivo nuclear magnetic resonance (NMR) measurements^41^ enable a three dimensional picture of lithium distributed in the brain in pharmacotherapy, but due to the method-associated high detection limit are not sensitive enough to measure the physiological endogenous lithium distribution. We have succeeded in developing a new technique (neutron-induced coincidence method “NIK”)^42^ for position sensitive, three dimensional (3D) spatially resolved detection of lithium traces in post-mortem tissue, allowing for the first time in lithium research, to collect a three dimensional high resolution map of the regional *trace* lithium content in the non lithium-medicated human brain.

## Results

139 samples of different brain regions in bilateral setting of 3 deceased individuals I–III (Table 1) were taken at autopsy at the “Institut für Rechtsmedizin” Munich, Germany (IRM).

**Table 1:** Brain sample collective, n_brain_ = 3, n_samples_ = 139.

Brain sample collective								
	Age/sex	Cause of death	Anamnestic data	Suicide (verified)	Lithium medication in death	Timespan death—autopsy	Samples	
							n	Regions
I	31/m	Pulmonary embolism^a^ (deep vein thrombosis)	No psychiatric illness	No	No	< 48 h	n = 43	– Frontal lobe – Temporal lobe – Occipital lobe (case II, III) – Brain stem – Basal ganglia (striatum, pallidum) – Limbic system (hippocampus, amygdala, thalamus, cingulate cortex, corpus callosum, fornix region)
II	43/m	Strangulation^a^	Depression, aggressive impulse control disorder	Yes	No	< 48 h	n = 46	
III	46/m	Barbiturate Intoxication^a^	Amyotrophic Lateral sclerosis Barbiturate prescription	No	Yes	< 48 h	n = 50	

^a^No pathomorphological signs of relevant brain edema, no macroscopic signs of degradation.

### Mean brain tissue concentration levels

The overall obtained concentration level (lithium area density λ, spatially resolved lithium concentration c_wet_Li_nat_) of trace lithium (Case I, II) is in the range of low ppb (λ: < 0.01–1.18 pg/cm^2^; c_wet_Li_nat_: 0.01–1.16 ng/g). The obtained concentration level of lithium in Case III (lithium medication) is in the medium range of ppb (λ: 68–231 pg/cm^2^, c_wet_Li_nat_: < 32–189 ng/g). In case I the range of spatially resolved lithium concentration c_wet_Li_nat_ is based on n = 29 out of n = 43. The results are shown in Table 2.

**Table 2:** Average brain lithium levels.

Average concentration levels						
	Lithium area density λ (pg/cm^2^)			Normalized spatially resolved lithium c_wet_Li_nat_ (ppb resp. ng/g)		
	Minimum	Median/Mean	Maximum	Minimum	Median/Mean	Maximum
I natural death (n = 43)	~ 0.04	0.38/0.46	1.18	0.02*	0.19*/0.23*	0.65*
II Suicide (n = 46)	~ 0.03	0.37/0.36	0.99	0.03	0.32/0.31	1.16
III Lithium medication (n = 50)	68	140.3	231	32	73.8	189

*Based on sample analysis of n = 29 of n = 43.

### Case-specific brain lithium distribution

#### Case I: no lithium medication; natural death, n = 43

**Figure 1:**
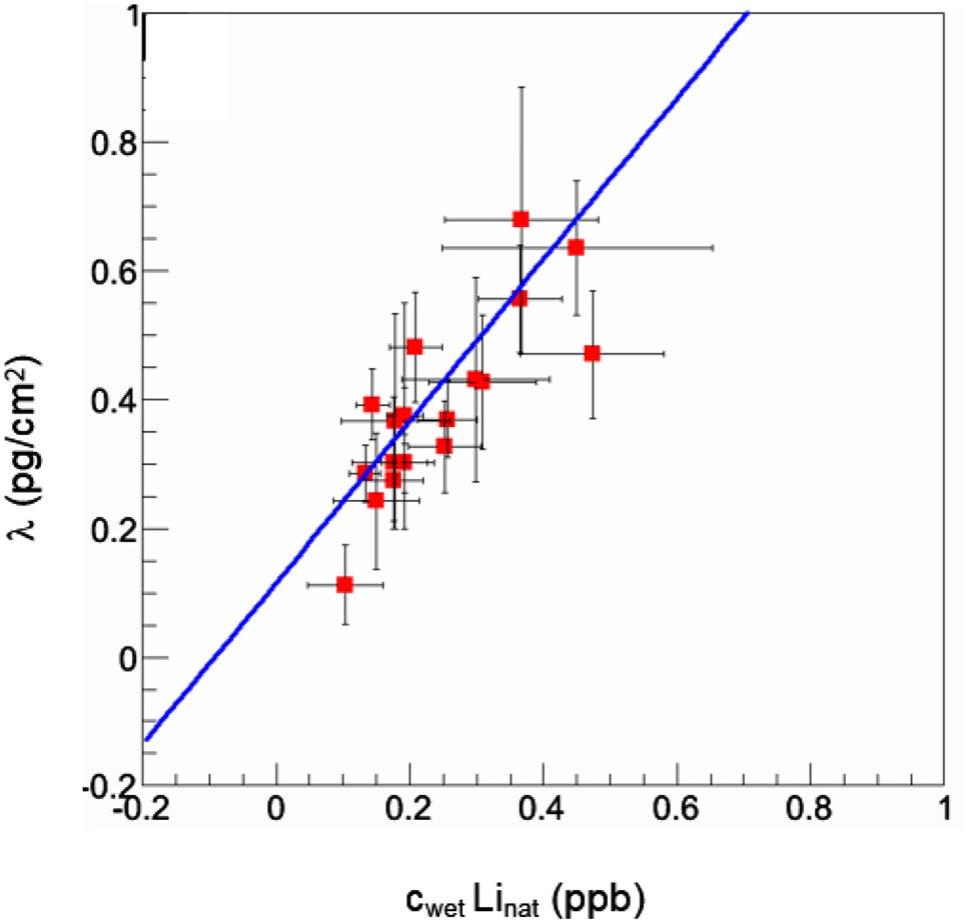
Case I: Correlation diagram c_wet_Li_nat_ and λ. The offset of the correlation fit indicates the level of the systematic uncertainty for the transformation from an areal density λ to a concentration in the native tissue.

In all 43 samples the lithium area density λ was measured. In correlation with the results of the layer thickness measurement available for n = 29 samples a high significant correlation between λ and c_wet_Li_nat_ (r = 0.809, p < 0.001) proves, that λ is sufficient for comparative assessments (Fig. 1) and the further analysis can be based on the results for λ in addition to the evaluation of c_wet_Li_nat_ (n = 29).

**Figure 2:**
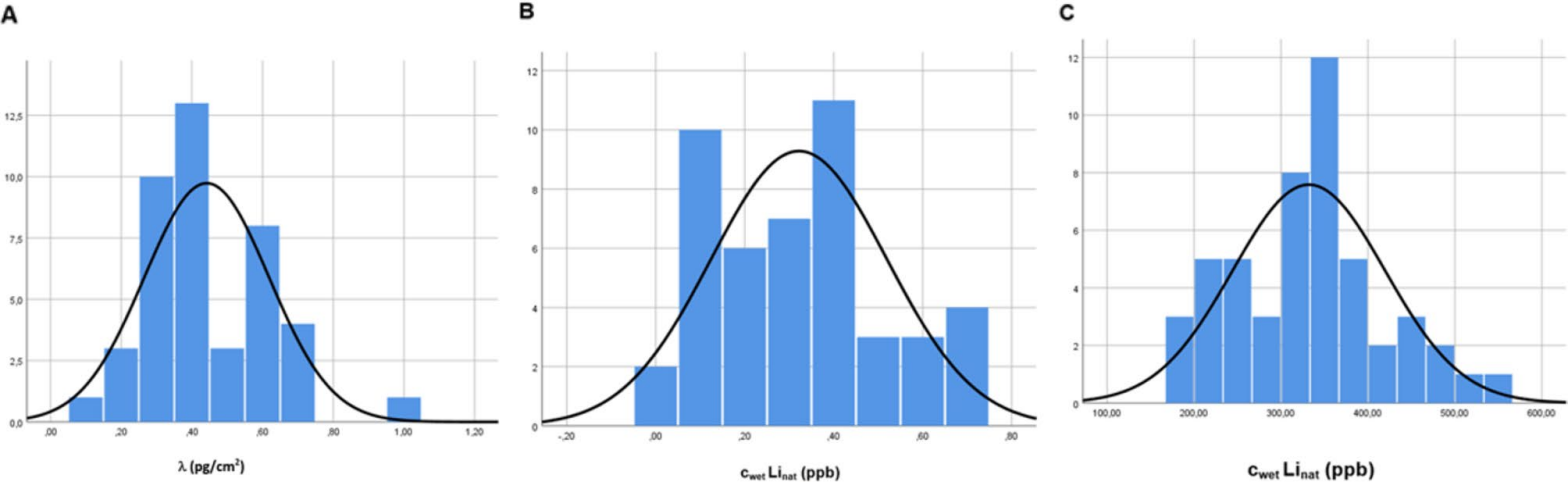
Histogram of lithium distribution in the brain* (**A**) case I; (**B**) case II; (**C**) case III. *Case A: lithium area density λ; Case II, III: spatially resolved lithium concentration c_wet_Li_nat_.

**Figure 3:**
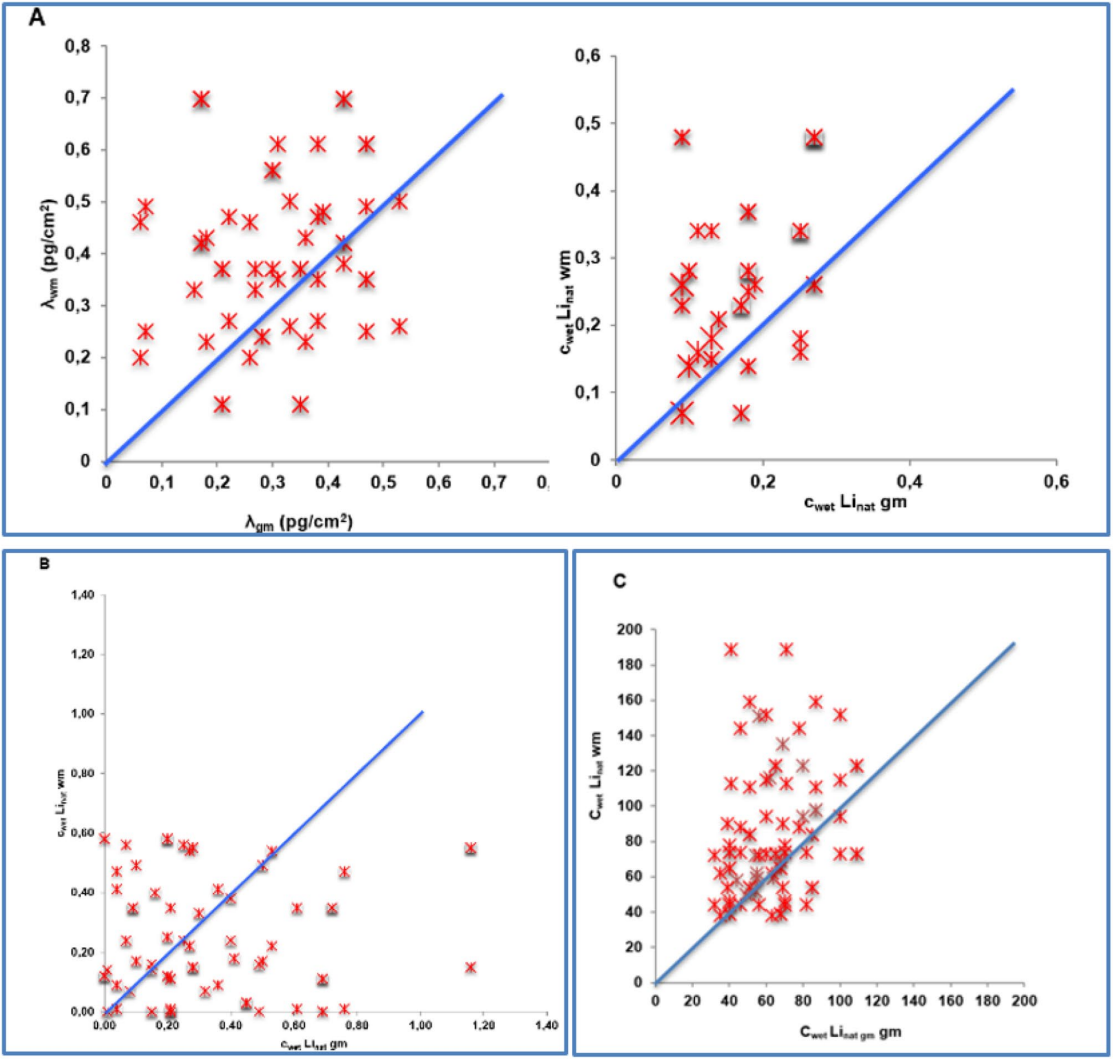
Correlation of lithium distribution in white versus gray matter (**A**) case I; (**B**) case II; (**C**) case III. *Case A: lithium area density λ; Case I–III: spatially resolved lithium concentration c_wet_Li_nat_.

The histogram of λ shows the anisotropic distribution in the examined brain regions by resembling a Gaussian distribution (Fig. 2A). Furthermore lithium concentration of λ (n = 43) proves a ratio well > 1 (1.34 ± 0.035) for white matter versus gray matter c_wm_/c_gm_. This can be further confirmed with a ratio well > 1 for c_wet_Li_nat_ (n = 29) correlating c_wm_/c_gm_ (Fig. 3A). Compared with other brain regions the lithium area density λ in the white matter of the thalamic region is with 0.99 ± 0.19 pg/cm^2^ significantly higher and shows the overall highest concentration in case I. On the other hand, the gray matter proportion of the subgenual part of the cingulate gyrus, the so-called Brodmann Area 25 (BA25) shows with λ: 0.64 ± 0.15 pg/cm^2^ and 0.45 ± 0.27 ppb the highest lithium concentration of the gray matter measurement. Except for the proportions of gray matter in the region “Amygdala” (0.6 pg/cm^2^ ± 0.15) comparatively high concentrations were only found in the white matter proportion. The regional results for case I are shown in Table 3.

**Table 3:** Regional lithium concentration, case I–III.

Regional Lithium concentration, case I–III												
	Case I, n = 43				Case II, n = 46				Case III, n = 50			
Region	Gray matter		White matter		Gray matter		White matter		Gray matter		White matter	
	λ (pg/cm^2^)	c_wet_Li_nat_ (ppb)	λ (pg/cm^2^)	c_wet_Li_nat_ (ppb)	λ (pg/cm^2^)	c_wet_Li_nat_ (ppb)	λ (pg/cm^2^)	c_wet_Li_nat_ (ppb)	λ (pg/cm^2^)	c_wet_Li_nat_ (ppb)	λ (pg/cm^2^)	c_wet_Li_nat_ (ppb)
Frontal lobe	0.43	0.31	0.33	−\−	0.45	0.40	0.53	0.38	68	44	77	50
	0.16	−\−			0.35	0.40	0.28	0.240.24	94	59	88	58
					0.54	0.51			71	49	142	96
											201	96
Temporal lobe (Insula region)	0.38	0.19	−\−	−\−	0.48	0.65	−\−	−\−	128	63	192	69
					0.22	0.14			189	68		
Occipital lobe	−\−	−\−	−\−	−\−	0.24	0.20	0.25	0.25	78	54	111	62
							0.05	0.05	80	55	130	72
									94	56	185	151
											187	114
Brain stem	0.27	−\−	0.37	−\−	0.52	0.56	−\−	−\−	−\−	−\−	−\−	−\−
Basal ganglia (Striatum, Pallidum)	0.38	0.19	0.56	0.37	0.22	0.14	0.45	0.33	128	63	187	98
	0.30	0.18	0.65	−\−	0.33	0.30	0.58	0.40	171	87	217	94
									201	8080		
**Limbic system**												
Brodman Area 25	0.64	0.45	0.11	0.10	0.10	0.09	−\−	−\−	96	64	105	59
					0.20	0.22			115	69	173	135
					0.39	0,38						
Cingulate cortex	0.37	0.18	−\−	−\−	Rostral: 0.22	Rostral: 0.21	−\−	−\−	Rostral: 201	Rostral: 80	−\−	−\−
					Medial: 0.69	Medial: 0.72			Caudal: 200	Caudal: 93		
					Dorsal: 0.60	Dorsal: 0.45						
					0.61	0.66						
					0.58	0.60						
					Caudal: 0.40	Caudal: 0.31						
					0.48	0.41						
Corpus callosum	−\−	−\−	Rostral: 0.68	Rostral: 0.37	−\−	−\−	Rostral: 0.40	Rostral: 0.35	−\−	−\−	Rostral: 231	Rostral: 123
			Medial: 0.43	Medial: 0.30			Medial: 0.34	Medial: 0.35			Medial: 201	Medial: 96
							Dorsal: ~ 0.03	Dorsal: ~ 0.03			Dorsal: 142	96
							Caudal: 0.20	Caudal: 0.18				
Thalamus	0.47	0.27	0.99	−\−	Rostral: 0.10	Rostral: 0.09	rostral: 0.40	Rostral: 0.35	122	62	211	116
					Medial: 0.16	Medial: 0.08	Medial: 0.15	Medial: 0.07				
					Dorsal: 0.60	Dorsal: 0.45	Dorsal: 0.03	Dorsal: 0.03				
Fornix region	0.30	0.18	0.56 0.65	0.37	0.69	0.72	0.34	0.35				
				−\−	0.52	0.56						
Hippocampus	Rostral: 0.28	Rostral: 0.13	Rostral: 0.24	Rostral: 0.15	Rostral: 0.63	Rostral: 0.37	Medial: 0.10	Medial: 0.07	Rostral: 89	Rostral: 55	Rostral: 110	Rostral: 59
	0.27	0.18	0.33	0.25	Medial: 0.43	Medial: 0.32			82	42	Dorsal: 192	Medial: 52
	0.39	0.14	Dorsal: 0.38	Dorsal: −\−	Dorsal: 0.47	Dorsal: 0.31			medial: 94	medial: 54	medial: 95	dorsal: 69
	Dorsal: 0.43	Dorsal: −\−	0.48	0.21					dorsal: 189	dorsal: 68	dorsal: 192	
	0.60	−\−										
	0.63	−\−										
Amygdala	Rostral: 0.28	Rostral: 0.13	Rostral: 0.24	Rostral: 0.15	0.47	0.31	−\−	−\−	Rostral: 89	Rostral: 55	Rostral: 110	Rostral: 59
	0.30	0.14	0.37	0.26					Medial:94	Medial: 54	Medial: 95	Medial:52
	0.39	0.19	Dorsal: 0.48	Dorsal: 0.21					Dorsal: 189	Dorsal: 68	Dorsal: 192	Dorsal: 69
	Dorsal: 0.60	Dorsal: −\−										
	0.63	−\−										

#### Case II: no lithium medication; suicide, n = 46

For all 46 samples λ and c_wet_Li_nat_ were measured. In case II the histogram of c_wet_Li_nat_ shows an anisotropic distribution as well, also resembling a Gaussian distribution (Fig. 2B).

Likewise in case I in case II c_wm_ versus c_gm_ of c_wet_Li_nat_ was correlated. Almost diametrically opposed to Case I the correlation diagram of c_wet_Li_nat_ in Case II shows a ratio well < 1 (0.71 ± 0.02) of c_wm_/c_gm_ (Fig. 3B). Here significantly more entries are below the bisector, indicating that there is significantly less lithium in white matter than in gray matter. Comparably high white matter concentrations show the rostral corpus callosum and the basal ganglia. Highest concentrations in gray matter are found in the medial and dorsal cingulate cortex, the fornix region and temporal lobe. The lowest values for white matter are measured in the dorsal corpus callosum, the occipital lobe, thalamic and hippocampal regions. The lowest values measured in the gray matter were in the anterior thalamic region and BA25. Comparably low concentrations were also found in the rostral cingulate cortex. The regional results for Case II are shown in Table 3.

#### Case III: lithium medication; n = 50

For all 50 samples λ and c_wet_Li_nat_ were measured. In case III the histogram of c_wet_Li_nat_ confirms the anisotropic distribution in turn by also resembling a Gaussian distribution (Fig. 2C). The correlation of c_wet_Li_nat_ c_wm_ versus c_gm_ showed as in case I with 1.44 ± 0.01 a ratio well > 1 (Fig. 3C). Beside high concentrations found in the occipital lobe, the regional highest lithium concentration of the white matter section are located at BA25, the rostral section of corpus callosum and the thalamic region. The concentration in the overall gray matter area was comparably homogeneous, only basal ganglia and cingulate gyrus showed slightly higher concentrations. The regional results for case III are shown in Table 3.

### Cross-case evaluation

**Figure 4:**
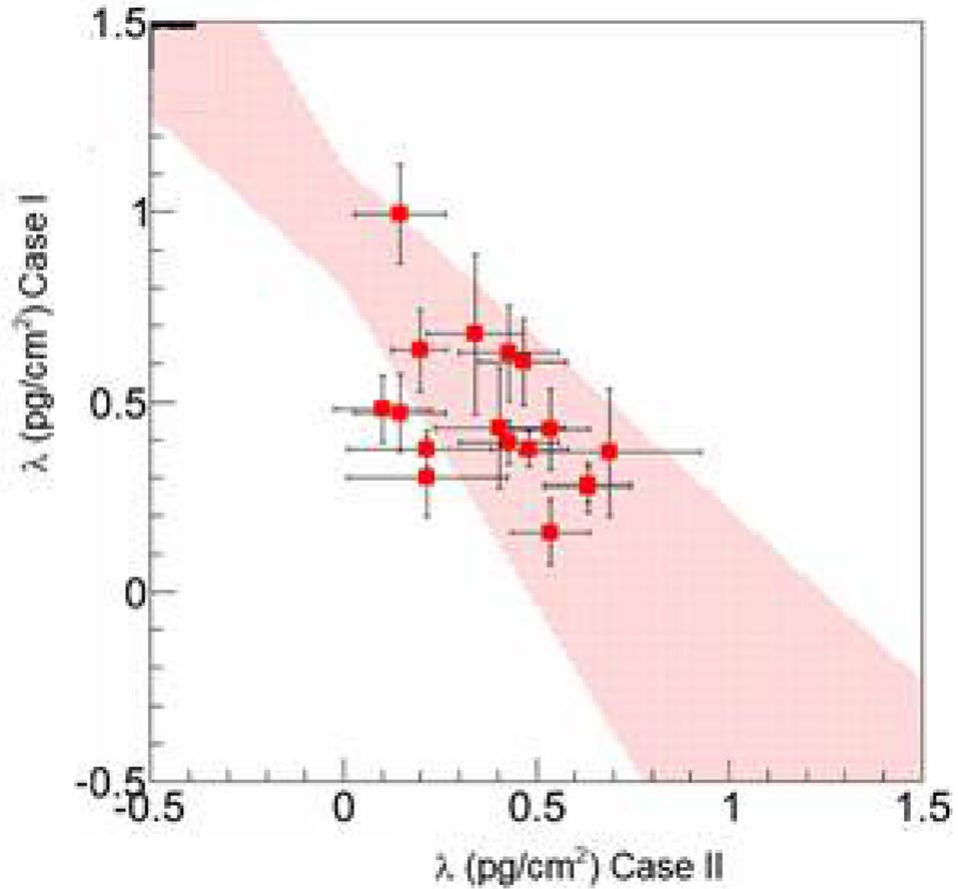
Correlation diagram of lithium area density λ_I_ versus λ_II_; *Pink colour* anticorrelation string.

With regard to any significance of trace lithium brain content for suicide a correlation of the results in case I and case II seems relevant. First, in case II the overall concentration is slightly lower than in case I. In average 17.5% of the case II samples less than 0,2 ppb lithium was measured, in case I only 5%. The correlation diagram (Fig. 4) of lithium area density λ_I_ to λ_II_ shows an anti-correlation with r = − 0.436 (p < 0.035). The anticorrelation string shows the fit of the measurement results.

## Discussion

The found average trace lithium concentration for case I and II was in the range of low ppb. According to MOORE et al., based on spectrometric measurements the ratio of the trace lithium content in tissue to blood plasma is in the range of c_tissue_/c_plasma_ 0.50–0.97^47^. Our results agree very well with that, taking also in account the found tissue distribution ratio and due to hemolytic changes under postautoptic conditions the necessity for whole blood determination. On the other hand, compared with case I and II, the average lithium concentration in case III with known lithium medication was only a factor > 200 higher, thus lower than therapeutic blood levels (4–8 ppm) and so lower than the aforementioned ratio c_tissue_/c_plasma_. An inference that the lithium medication was discontinued shortly before death therefore would seem plausible. Taking into account, that abruptly stopping lithium medication without gradual titration triggers suicidal ideation^48^ gives also a hint, that the lethal barbiturate intoxication in case III (progredient ALS, existent barbiturate prescription, no documented suicidal ideation) may have been non accidental, but with suicidal intent. The results show an anisotropic regional lithium brain distribution in all three cases, both without and with lithium medication. They correlate well with data for trace lithium concentrations in rodent brain^49^ and also NMR-Studies on lithium medicated humans^41^.

The histogram of lithium distribution in the different brain regions of all cases approaches a Gaussian distribution, thus indicating a homeostatic regulation under endogenous and also medicated conditions. As a normal (rather than log-normal) distribution of an element’s tissue concentration is considered as criterion for essentiality^50^, our results accordingly support the thesis that lithium may be of essential relevance not only in animal, but also in man^15^.

Another essential finding from the measurements emerges when considering the differences in distribution between white and gray matter. In Case I and III there was a clear difference in the lithium concentration in favor of white matter with a ratio for cLi_wm_/cLi_gm_ well > 1, which allows a conclusion that the core areas contain significantly less lithium than the axonal components. Similar results with regard to an imbalance in the distribution of lithium in between white and gray matter in the lithium medicated brain have been found in animal experiments, where lithium supplemented rats showed two to three times higher lithium concentrations in white matter than in gray matter^51^. A possible explanation for this may be, that in the axon the density of voltage-sensitive lithium-permeable sodium channels is higher. This would enable intraneuronal lithium concentrations to vary depending on the action potential frequency^52^. And as the potential of lithium at equilibrium is less negative in regions with high myelin density (→ axon) than in regions with a low myelin density (→ core region) the presence of myelin would thus reduce a sodium channel-associated passive diffusion of lithium with a dampening effect on brain excitability^53^. However, in case II (suicide) the ratio for cLi_wm_/cLi_gm_ was well < 1, further proved by an anticorrelation of overall λ_I_/λ_II_ (Fig. 10). An explanation thesis for this almost diametrical distribution imbalance would be on the basis of the above statements that based on excitatory events on the axon membrane a redistribution of lithium via sodium channels out of the myelinated area occurs in the immediate run-up to the suicidal event. Another explanation could hint to a primary, i.e. an already existing lithium deficiency or a “utilization disorder”. Although the extent to which a primarily higher endogenous concentration of lithium in the white matter of the human brain compared to gray matter could mean a mood-stabilizing and thus possibly antisuicidal effect, remains speculative to date.

Another interesting result is the distributional imbalance in the so called emotional regions, in particular Thalamus and BA25. Both brain regions are part of the limbic system and of exceptional relevance with regard to affect-regulating processes. In Case I the tracts of the thalamic region contained significant more lithium than any other regions with a ratio cLi^Th^_wm_/cLi^Th^_gm_ of approximately 2. In case III also the white matter concentration of the thalamic region was comparably high with a ratio cLi^Th^_wm_/cLi^Th^_gm_ of approximately 1.8. In Case II the rostral part showed a much higher concentration in the tracts and a very low concentration in the core section (cLi^Th^_wm_/cLi^Th^_gm_ ca. 3.5). The medial parts of the thalamic tracts as much as the core section showed comparably low lithium concentrations with a ratio cLi^Th^_wm_/cLi^Th^_gm_ = 1. And the dorsal part showed a high concentration in the core section, but very low concentration in the tracts (cLi^Th^_wm_/cLi^Th^_gm_ approx.. 0.1), indicating an innerthalamic lithium distribution imbalance in suicide versus control. The thalamus is a structure consisting of numerous different core complexes as well as somatosensitive and motor fiber connections and seems to play an essential role as a switching point in mood regulation and the genesis of affective disorders^54^. Ischemia-related lesions of the dorsomedial thalamic nuclei can cause a transient maniform syndrome^55^. Furthermore, the thalamic volume appears to increase with lithium medication^56^ and the thalamus volume during lithium-treatment of patients with bipolar affective disorder increases compared to non-lithium-treated patients^57^. This indicates a structural importance of lithium in the thalamic metabolism.

The subcallous or subgenual part of the anterior cingulate gyrus BA25 consists of cortical tissue with adjacent tracts. It constitutes an important node in the limbic system network, e.g. thalamic structures and seems to play an important role in depressive disorder^58^. SASSI et al. showed in a study on bipolar patients, that untreated patients compared to lithium-treated patients and controls had a lower volume of the anterior cingulate gyrus^59^, assuming a lithium-associated structural benefit of lithium. MAYBERG et al. demonstrated, that this region, which is metabolically over reactive in therapy-resistant depression, is positively influenced by deep brain stimulation of the white matter adjacent to the area^60^. Our results in cases I and II show comparably high lithium levels in BA25 compared to other brain areas. In case I the cortical structures of BA25 showed the highest lithium concentration of all examined core structures, while the adjacent white matter lithium content was comparably low. In case III BA25`s overall lithium content was comparably high with an almost balanced lithium content in white vs gray matter (ratio cLi^BA25^_wm_/cLi^BA25^_gm_ approx. = 1). But in case II the lithium concentration in the cortical part of the BA25 went to zero, while showing a medium range in the adjacent tracts, suggesting an almost diametral distribution imbalance in suicide versus control.

### Limitations

The lithium distribution patterns so far obtained with the NIK method, thus in no way contradicting given literature references, are based on post mortem tissue. Although no macroscopic signs of degradation were observed, the extent to which an element-associated post-mortem redistribution at the molecular level—also in view of the cryopreservation procedure—may have caused significant discrepancies in relation to the vital state, cannot be methodologically cross-checked. Also a potential influence by certain agonal phenomena—even if there was no higher-grade cerebral edema in particular in case II (suicide by hanging)—cannot be excluded with certainty and therefore needs to be checked on a larger collective. Another limitation arises from sample collective limitations, selections and restrictions on the basis of the tissue law and ethical considerations.

## Conclusion

With the use of the NIK method, it has been possible for the first time to map the distribution pattern of the endogenous lithium content in the various regions of the human brain. The so found intra- and interindividual lithium distribution in the brain is not only anisotropic under both endogenous and medicinal conditions, but also approximates a so-called Gaussian distribution. This speaks in favor of a homeostatic regulation of both endogenous and drug-induced lithium concentrations and could serve as an indication for the essentiality of the trace element. In case of suicide the ratio of white matter versus gray matter lithium concentration was < 1 and thus almost diametral to the control collective, where the ratio was well > 1, so that an acute lithium depletion in suicide could be considered. In addition, in the so-called emotional regions thalamus and BA25 noticeable lithium distribution difference between suicide and control collective were detected. Due to the small number of cases, the quality of information is limited and should be checked on a larger collective.

## Methods

The authors have further established and applied a hitherto unique measurement method to detect and spatially resolve even smallest amounts of trace lithium in organic samples for examining different brain regions in suicide versus control. Numerous samples of multiple brain regions in bilateral setting (n = 139) of 3 deceased individuals (Table 1) were taken at autopsy at the “Institut für Rechtsmedizin” Munich, Germany (IRM).

The experiments were conducted at the research reactor “Forschungs-Neutronenquelle Heinz Maier-Leibnitz” (FRM II) and the Prompt Gamma Activating Analysis (PGAA) in Garching/Munich, Bavaria, where a well focused beam of cold neutrons with a neutron flux of ɸ = 1.2 × 10^10^ cm^−2^ s^−1^ is available.

### Methodological background

**Figure 5:**
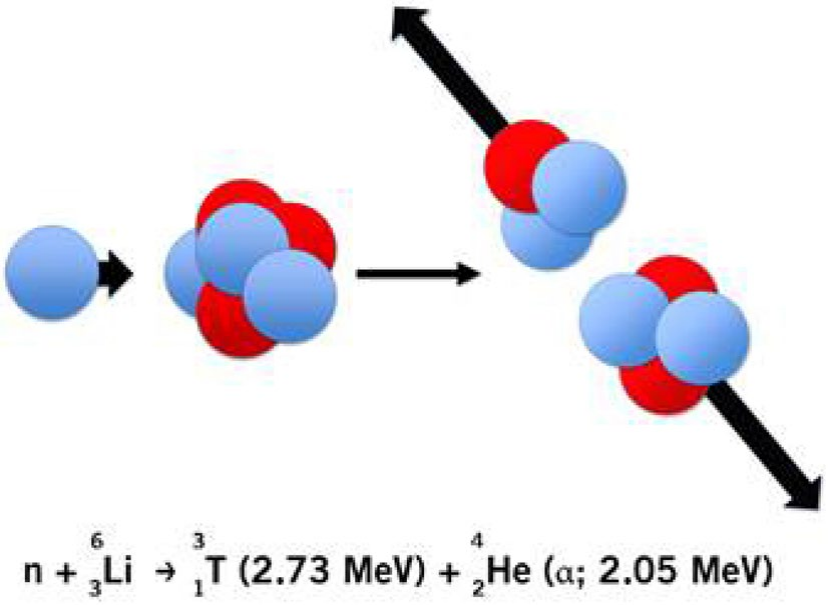
Schematic illustration of the ^6^Li(n,α)^3^H-reaction.

Lithium exists in nature as a mixture of two stable Isotopes ^6^Li and ^7^Li in a fixed ratio of 7.59 ± 0.04% ^6^Li to 92,41 ± 0.04% ^7^Li^43^. The ^6^Li-Isotop exhibits an exceptionally large neutron capture cross section (E_n_ = 25 meV) for thermal neutrons of σ_n_ = 940 ± 4 b, scaling for cold neutrons with E_n_ = 1.83 meV according to the optical theorem to σ_n_ = 3474 b^44^. After the capture of a neutron, its nucleus is highly excited so that particles fission almost instantaneously into an alpha (^4^He)- and a tritium (^3^H) particle (see Fig. 5 for illustration).

**Figure 6:**
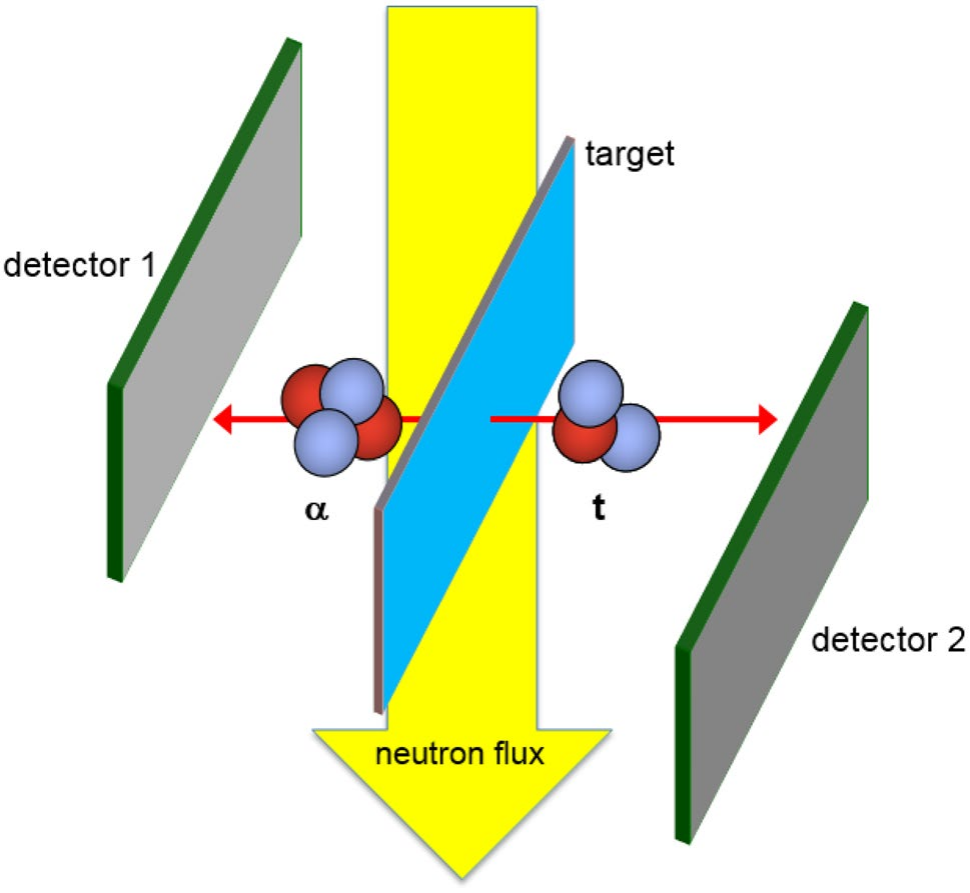
Experimental concept for the position sensitive lithium detection with the ^6^Li(n, α)^3^H-reaction. Both 20 × 20 mm^2^ large silicon detectors have resistive layers for charge splitting on both surfaces. Two readout strips at the edges in horizontal and vertical direction respectively allow for a position resolution of σ_x,y_ ~ 0.2 mm for a typical particle energy of 2 MeV.

The dominant reaction channel ^6^Li(n,α)^3^H with the two particles in its exit channel obtains a fixed energy of E_3H_ = 2.73 meV and E_α_ = 2.05 meV. With the center of mass system being nearly at rest in the laboratory frame, ^4^He and ^3^H are emitted at an angle of Θ = 180° with respect to each other. The coincident detection of the kinetic energy and the impact sites of both particles via detector measurement enables the retrograde reconstruction of the reaction site inside the sample (see Fig. 6). The associated coincident energy pattern is unique, as the total energy deposit in the detectors is far above the energy signals by the dominant background radiation (β and γ particles) or a small branch particle background from the ^10^B(n,α)^7^Li reaction with about 2 meV less kinetic energy distributed on both reaction products. But especially the time coincidence measurement of signals in both detectors with a time difference ∆t < 200 ns provides an extreme selectivity for the detection of ^6^Li.

**Figure 7:**
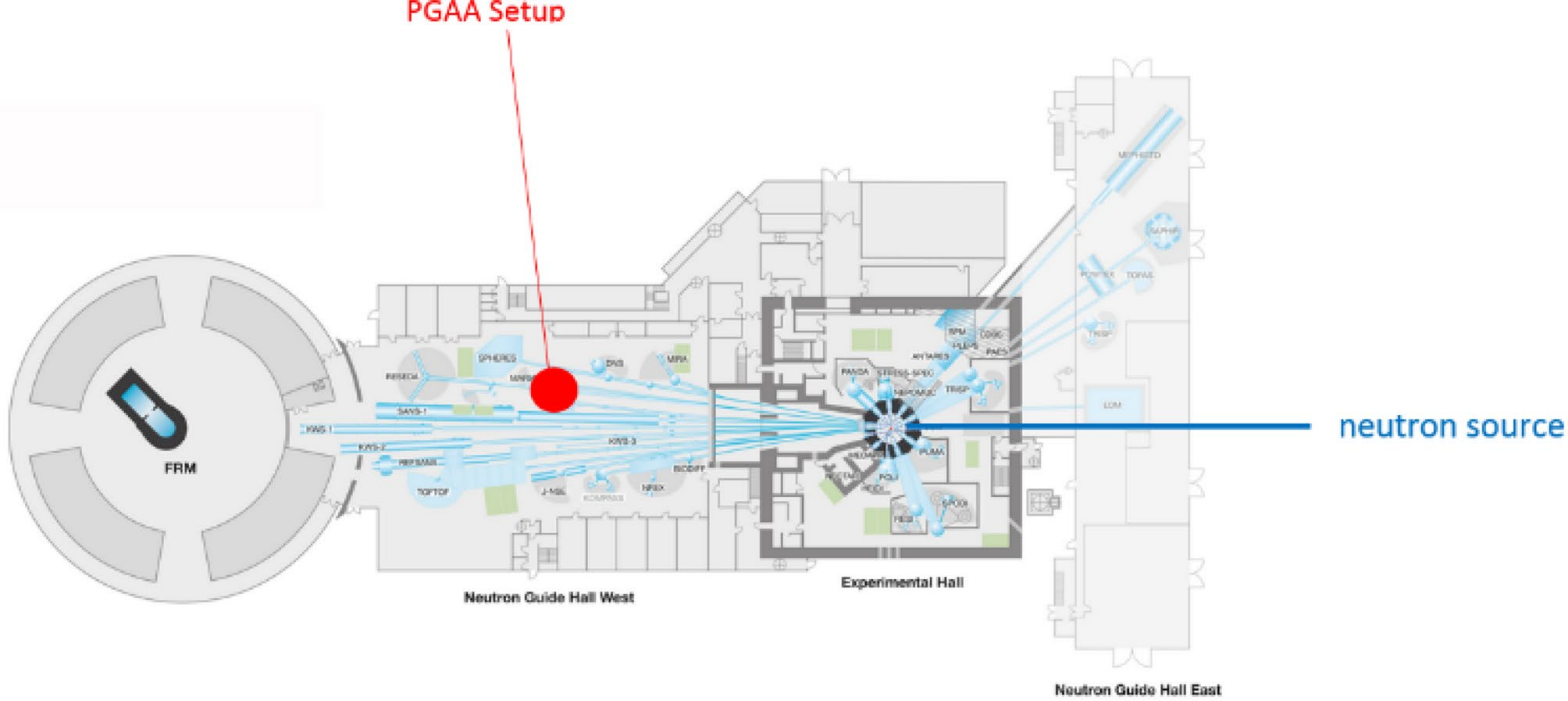
Reactor assessment FRM II, schematic overview. mod. n. MLZ Overview: Lageplan_2013_All_engl; http://www.mlz-garching.de/instrumente/bildgebende-verfahren-und-analyse.html. Accessed 01 October 2020.

**Figure 8:**
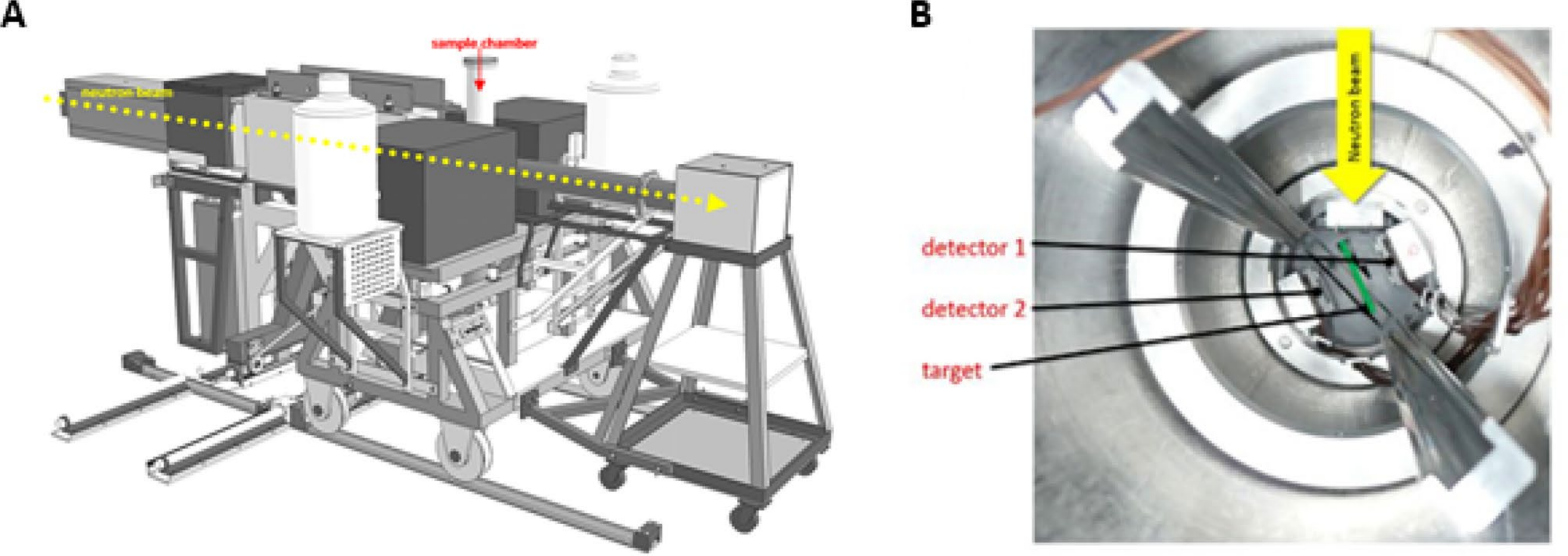
Prompt Gamma Activation Analysis (PGAA): (**A**) schematic overview; (**B**) sample chamber.

The flow of cold neutrons is released from the reactor guided via neutron mirrors through vacuum pipes to the PGAA target point (Fig. 7). The sample chamber of the PGAA measurement station consists mainly of Aluminum (Al) and Polytetrafluorethylen (Fig. 8). These are materials with very small neutron capture cross-sections and daughter nuclei with very short lifespans. The measurement setup consists of a compact detector arrangement with a collimator for the neutron flux in the front, two position sensitive silicon detectors on both sides mounted diametral to each other and tilted by Θ = 25° with respect to the axis of the neutron beam to optimize the solid angle coverage and a sample holder in the middle (see Fig. 6). The sample holder with a size of 40 mm × 30 mm × 0.2 mm was designed to guarantee a maximum overlap between the collimated neutron beam and the sample, but no structural elements in the area of the neutron flux. The sample holder consists of a pure aluminum frame (cAl > 99.999%) covered with foil of lithium-free Polyethylene (PE) with a calibrated thickness of 0.9 ± 0.2 μm and is prepared with a layer of neutralizing bipolar ions (RI 65 P, Ringionisator, Haug) to avoid possible contamination by dust particles.

### Sample preparation

**Figure 9:**
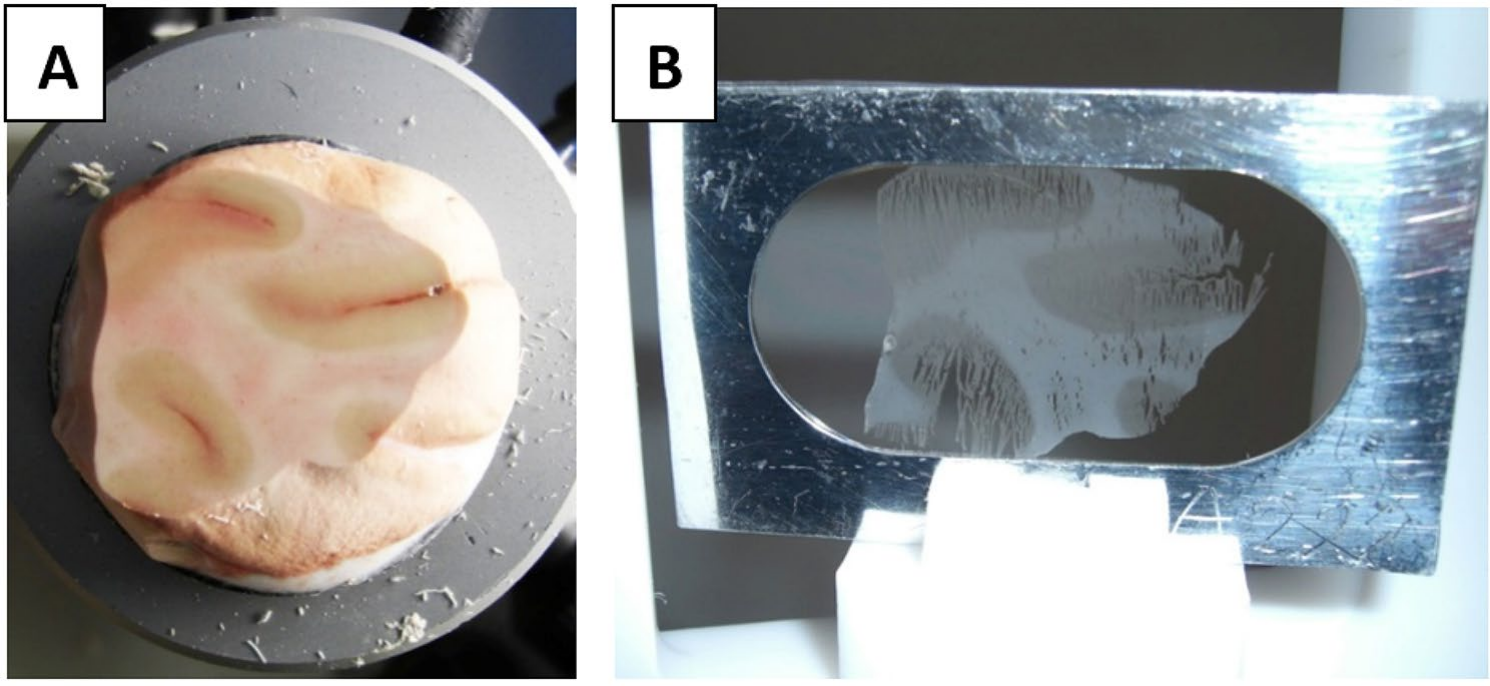
Cryotome brain sample: (**A**) sample on cryotome holder; (**B**) 10 µm slice attached to the sample frames made from a self-supporting d = 0.9 µm polyethylene cling film, fixed to an outer 300 μm thick aluminum target frame.

After the extraction of approximately 2.5 × 2.5 × 1.5 cm^3^ large brain samples and storage in polyethylene bags at a temperature of − 20 °C they were sliced with a cryotome at the IRM without interrupting the cold chain. Because of limitations by the energy loss of particles passing through matter with an maximum range of 1.3 mg/cm^2^ for the alpha-particles, a sample thickness of d = 10 μm was chosen. The slices were then centered on the substrate foil of the sample frame (see Fig. 9A,B). To minimize dust contaminations on the support foils of the sample frame fabrication was done in a clean room. An accessory slice was put on a microscopic slide for further neuropathological assessment. After each slice section the next (n ≤ 10) slices made were discarded to minimize destructive temperature influences on the sample surface. For further reduction of the sample mass layer and guarantee a long term stability the cryotome sample then is lyophilized for 8 h in vacuum and conserved in a lithium-free PE container before PGAA measurement. To control contamination of the samples, empty sample frames were treated in parallel with identical procedures and stored in each box for reference.

### Neuropathological assessment

The additionally obtained microscopic samples were stained with hematoxylin-and-eosin technique and examined with focus on brain region and tissue texture resp. discriminating regional white matter and gray matter structures.

### Layer thickness measurement

Lithium coverage of a cryosection sample depends on the respective sample thickness and varies significantly because of artifacts caused by cutting e.g. resulting in micro-cracks. Therefore a layer thickness measurement of every sample was applied. Every PGAA sample was bombarded with monoenergetic alpha-particles of an ^241^Americium (Am)-Source at a distance of 10 cm and the energy loss was measured with the same type of position sensitive silicon detectors as used in the NIK setup, which recorded the energy of the alpha-particles right after the sample. Taking into account of the “stopping power” amount defined for brain tissue^45^ the effective spatially resolved mass occupancy of the sample could be calculated.

### Calibration and background measurement

Reference samples with a known homogeneous lithium thickness and empty target holders were used to calibrate the detector setup and to evaluate the position resolution.

### Analytical controls

For all three cases additional measurement of brain tissue via atomic absorption spectroscopy (AAS) were conducted, analytically confirming the concentration range.

### Data analysis

Statistic data analysis was carried out with IBM SPSS Statistics Software, Microsoft Excel Mac OS 2011, Vers. 23 and “ROOT” (Extention of C++/LHC Cern).

**Figure 10:**
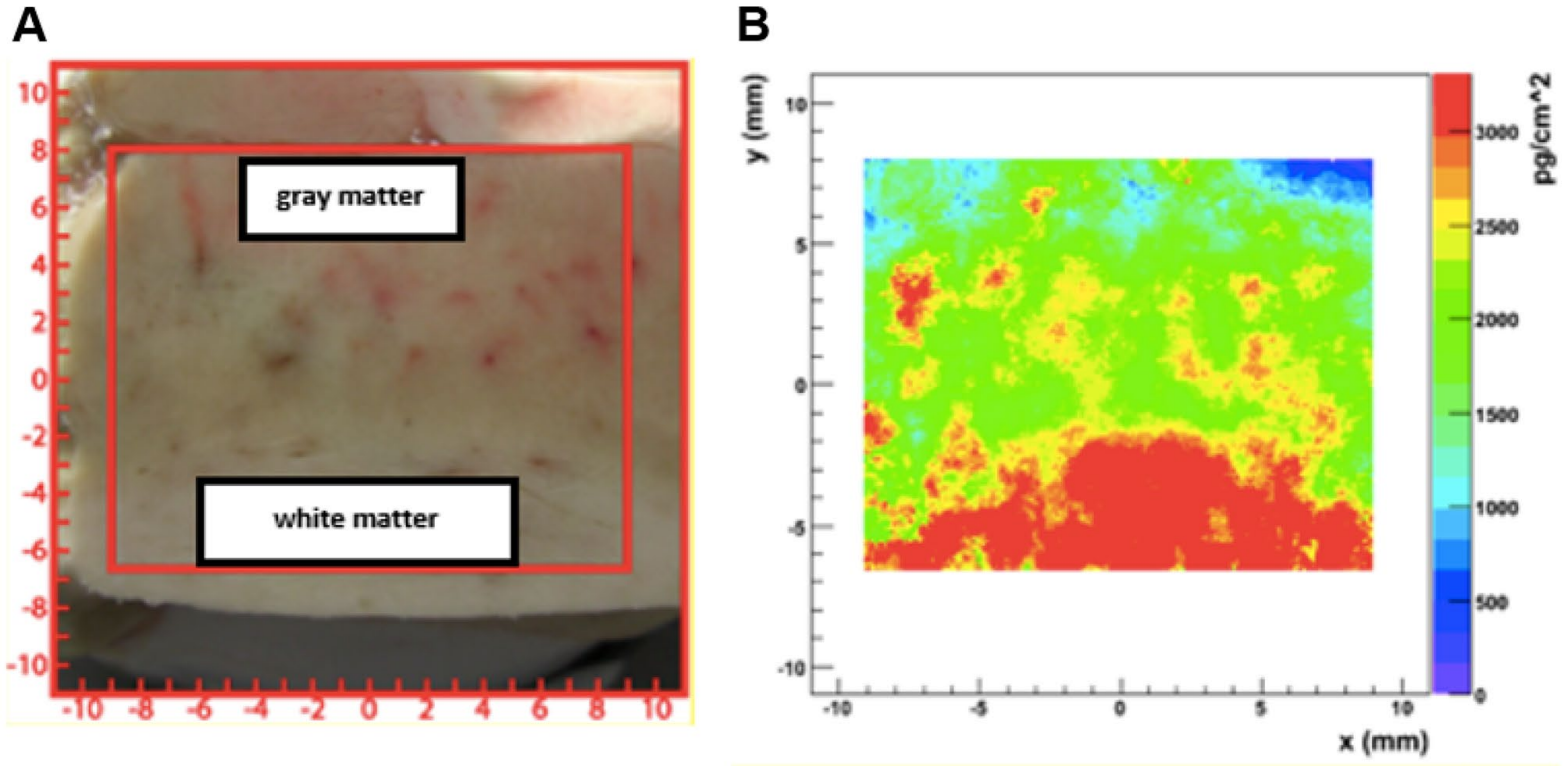
NIK Measurement result: (**A**) cryotome sample* (**B**) Lithium area density λ. For the position reconstruction, including the particle straggling a typical resolution of σ_x,y_ ≈ 0.4 mm was achieved. *Comparative sample: frontal lobe (lithium intoxication).

Using two hit points in the detector planes and the assumption of a planar target plane the emission points were reconstructed according to the theorem on intersecting lines. Hitting the detector, the particle’s impact energy is converted into an electrical charge collected in preamplifiers, converted into a voltage equivalent signals, digitized and stored on a hard drive. After data calibration and subtraction of background effects the lithium area density λ (pg/cm^2^) was obtained (Fig. 10). After normalization of λ with the local layer thickness of the sample the Lithium concentration c_dry_^6^Li could be calculated and by scaling with the average water content of brain structures^46^ and a three dimensional ^6^Li concentration in tissue c_wet_^6^Li was obtained. In the last step, with consideration of the natural lithium isotope ratio, the conversion in Li_nat_ resulted in the substrate morphometric documentation of the spatially resolved lithium concentration c_wet_Li_nat_ (ppb resp. ng/g).

### Detection and quantification limit

The currently possible quantification limit for _nat_Li with NIK is 60 fg/cm^2^ resp. 60 pg/g_wet_. The detection limit could be fixed with 8 fg/cm^2^ resp. 8 pg/g_wet_.

### Ethical approval

The study is approved by the ethics committee of the Ludwig-Maximilian-University Munich LMU (Nr. 300-10). The study was conducted in concordance with the human tissue act 2004/23/EG and the 59th WMA General Assembly Seoul 2008. The informed consent of the Legally Authorized Representative (LAR) and/or the next of kin of all deceased individuals whose brain tissue was studied was given prior to examination and data anonymization in accordance with the votum of the ethics committee of the Ludwig-Maximilian-University Munich LMU (Nr. 300-10). The study was performed in accordance with the ethical standards of the Declaration of Helsinki (1964) and its subsequent amendments.

## Abbreviations

AAS: Atomic absorption spectroscopy
Al: Aluminium
Am: Americium
BA25: Brodmann area 25
Ca^++^: Calcium cation
c_wet_Li_nat_: Normalized spatially resolved lithium
c_wm_/c_gm_: Lithium concentration in white versus gray matter
FRM II: Forschungs-Neutronenquelle Heinz Maier–Leibnitz
ICP-MS: Inductively coupled plasma mass spectrometry
IRM: Institut für Rechtsmedizin München
lambda λ: Lithium area density
Li^+^: Lithium cation
Li^+^/d: Daily uptake of lithium cations
Li^+^/oral unit: Lithium cation uptake per oral unit of pharmaceutical products
Mg^++^: Magnesium cation
n: Number of samples
NIK: Neutron-induced coincidence method
NMR: Nuclear magnetic resonance
PE: Polyethylene
PGAA: Prompt gamma activating analysis
ppb: Parts per billion
3D: Three dimensional
